# Quantitative MRI radiomics in the prediction of molecular classifications of breast cancer subtypes in the TCGA/TCIA data set

**DOI:** 10.1038/npjbcancer.2016.12

**Published:** 2016-05-11

**Authors:** Hui Li, Yitan Zhu, Elizabeth S Burnside, Erich Huang, Karen Drukker, Katherine A Hoadley, Cheng Fan, Suzanne D Conzen, Margarita Zuley, Jose M Net, Elizabeth Sutton, Gary J Whitman, Elizabeth Morris, Charles M Perou, Yuan Ji, Maryellen L Giger

**Affiliations:** 1 Department of Radiology, The University of Chicago, Chicago, IL, USA; 2 Program of Computational Genomics & Medicine, NorthShore University HealthSystem, Evanston, IL, USA; 3 Department of Radiology, University of Wisconsin—Madison, Madison, WI, USA; 4 National Cancer Institute, Cancer Imaging Program, Bethesda, MA, USA; 5 Department of Genetics, University of North Carolina—Chapel Hill, Chapel Hill, NC, USA; 6 Department of Medicine, The University of Chicago, Chicago, IL, USA; 7 Department of Radiology, University of Pittsburgh, Pittsburgh, PA, USA; 8 Department of Radiology, University of Miami Health System, Miami, FL, USA; 9 Department of Radiology, Memorial Sloan Kettering Cancer Center, New York, NY, USA; 10 Department of Radiology, MD Anderson Cancer Center, Houston, TX, USA; 11 Department of Public Health Sciences, University of Chicago, Chicago, IL, USA

## Abstract

Using quantitative radiomics, we demonstrate that computer-extracted magnetic resonance (MR) image-based tumor phenotypes can be predictive of the molecular classification of invasive breast cancers. Radiomics analysis was performed on 91 MRIs of biopsy-proven invasive breast cancers from National Cancer Institute’s multi-institutional TCGA/TCIA. Immunohistochemistry molecular classification was performed including estrogen receptor, progesterone receptor, human epidermal growth factor receptor 2, and for 84 cases, the molecular subtype (normal-like, luminal A, luminal B, HER2-enriched, and basal-like). Computerized quantitative image analysis included: three-dimensional lesion segmentation, phenotype extraction, and leave-one-case-out cross validation involving stepwise feature selection and linear discriminant analysis. The performance of the classifier model for molecular subtyping was evaluated using receiver operating characteristic analysis. The computer-extracted tumor phenotypes were able to distinguish between molecular prognostic indicators; area under the ROC curve values of 0.89, 0.69, 0.65, and 0.67 in the tasks of distinguishing between ER+ versus ER−, PR+ versus PR−, HER2+ versus HER2−, and triple-negative versus others, respectively. Statistically significant associations between tumor phenotypes and receptor status were observed. More aggressive cancers are likely to be larger in size with more heterogeneity in their contrast enhancement. Even after controlling for tumor size, a statistically significant trend was observed within each size group (*P*=0.04 for lesions ⩽2 cm; *P*=0.02 for lesions >2 to ⩽5 cm) as with the entire data set (*P*-value=0.006) for the relationship between enhancement texture (entropy) and molecular subtypes (normal-like, luminal A, luminal B, HER2-enriched, basal-like). In conclusion, computer-extracted image phenotypes show promise for high-throughput discrimination of breast cancer subtypes and may yield a quantitative predictive signature for advancing precision medicine.

## Introduction

Breast cancer is the most commonly diagnosed cancer among women in North America, and it is the second leading cause of cancer death in women.^1^ On the basis of receptor status, breast cancer can be classified into different subtypes. The three clinically most-useful receptors in breast cancer cells, because they dictate therapy, are the estrogen receptor (ER), progesterone receptor (PR), and human epidermal growth factor receptor 2 (HER2). HER2-positive breast cancers tend to be more aggressive and have a poorer prognosis than HER2-negative cancers, and ER-positive and PR-positive cases have lower risks of mortality compared with women with ER-negative and/or PR-negative disease.^2–5^ Triple-negative (TN) cases (negative for all three receptors) relapse more quickly, and thus account for a large portion of breast cancer deaths after diagnosis.^2–4,6,7^ By considering gene expression measurements, breast cancer can be categorized into several molecular subtypes, such as normal-like, luminal A, luminal B, HER2-enriched, and basal-like. Different molecular and receptor characterized subtypes have different prognoses and respond differently to specific therapies.^8–12^

An important project facilitating research in the molecular and genetic landscape of breast cancer is The Cancer Genome Atlas (TCGA), sponsored by the National Cancer Institute (NCI) and National Human Genome Research Institute.^13,14^ The Cancer Imaging Archive (TCIA) project is the imaging counterpart of the TCGA and has the goal to promote cross-disciplinary research to better understand the relationship between imaging phenotypes (i.e., radiomics) and genomic markers (i.e., genomics).^13,14^ Various investigators have been developing computerized image analysis for computer-aided diagnosis and the quantitative characterization (i.e., radiomics) of breast cancers on clinical images.^15,16^ It is important to understand the difference between ‘radiomics’ and ‘radiogenomics’.^17^ ‘Radiomics’ refers to the high-throughput extraction of quantitative features from images, i.e., conversion of images to mineable data, and subsequently using these data for decision support, including patient outcome. ‘Radiogenomics’ or ‘imaging genomics’ refers to the study of the associations between radiomic data (imaging features) and genomic patterns. It should be noted that radiogenomics is also used in the field of radiation oncology to refer to the study of genetic variation associated with effects from radiation therapy. Radiomics in breast cancer research is being conducted for the diagnostic differentiation of malignant from benign tumors as well as for prognosis in terms of invasiveness—identifying ductal carcinoma *in situ* and invasive ductal carcinoma, pathologic stage, lymph node involvement, molecular subtypes, and genomics.^18–30^ Bhooshan *et al.*^19,20^ related dynamic contrast-enhanced magnetic resonance imaging (DCE-MRI) radiomic features to breast cancer invasiveness and cancer type. Agner *et al.*^22^ extracted image-based features and used them to differentiate the TN cancers from other molecular subtypes. Mazurowski *et al.*^24^ analyzed 48 TCIA breast MRI cases, and related MRI enhancement dynamics to the luminal B subtype. Grimm *et al.*^25^ discovered relationships between MRI imaging features and luminal A and luminal B cancer molecular subtypes. Yamamoto *et al.*^26,27^ showed the association between radiogenomic biomarkers with early metastasis. Ashraf *et al.*^28^ found that intrinsic imaging phenotypes correlated with risk of recurrence score defined by Oncotype DX. Yamaguchi *et al.*^29^ studied the relationship between heterogeneous kinetic curve pattern and molecular subtype. Blaschke *et al.*^30^ reported that HER2-positive tumors have a greater portion of their tumor with rapid uptake compared with other molecular subtypes. In this paper, we demonstrate, for biopsy-proven invasive breast cancers, the relationships between molecular classifications of breast tumors, in terms of ER, PR, HER2, TN, and intrinsic molecular subtypes as determined from PAM50 microarray assay, and MRI phenotypes. We aim to provide a quantitative MRI-based signature to clinicians for assessing the prognosis of breast cancer and potentially the patient treatment strategy in personalized medicine.

## Results

**Figure 1:**
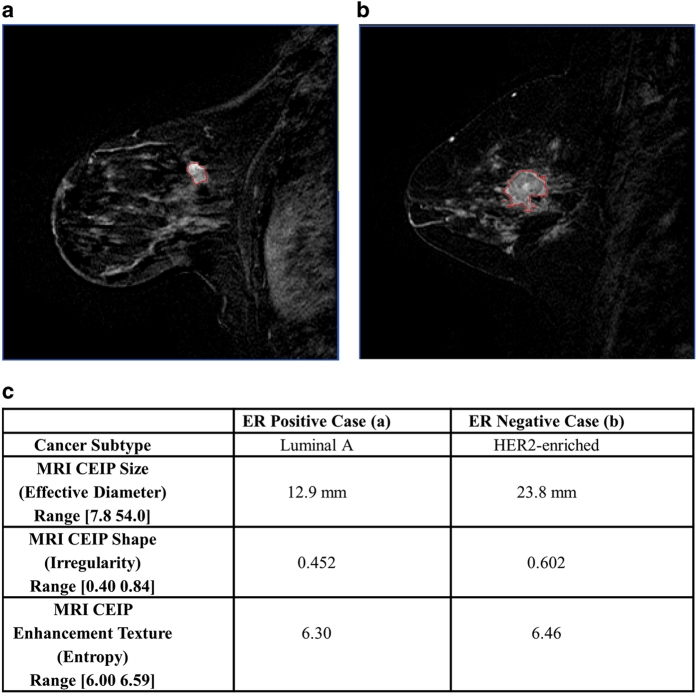
Example cases including segmentation outlines obtained from the computer segmentation method. (**a**) ER-positive example; (**b**) ER-negative example; (**c**) CEIP values (and ranges) for size, irregularity, and enhancement texture for two example cases. CEIP, computer-extracted image phenotypes; ER, estrogen receptor; HER2, human epidermal growth factor receptor 2; MRI, magnetic resonance imaging.

From receiver operating characteristic (ROC) analysis, the performance of the MRI phenotypic signatures in the task of distinguishing between clinical receptor status yielded area under the ROC curve (AUC) values of 0.89 (*P*-value <0.0001), 0.69 (*P*-value=0.0112), 0.65 (*P*-value=0.0491), and 0.67 (*P*-value=0.0404) in the tasks of distinguishing between ER+ versus ER−, PR+ versus PR−, HER2+ versus HER2−, and TN versus all others, respectively. Example cases, one ER-positive tumor and one ER-negative tumor, with tumor segmentation outlines from our computerized lesion segmentation method, are shown in Figure 1, along with computer-extracted image phenotype (CEIP) values (and ranges) for size, irregularity, and contrast enhancement heterogeneity.

**Figure 2:**
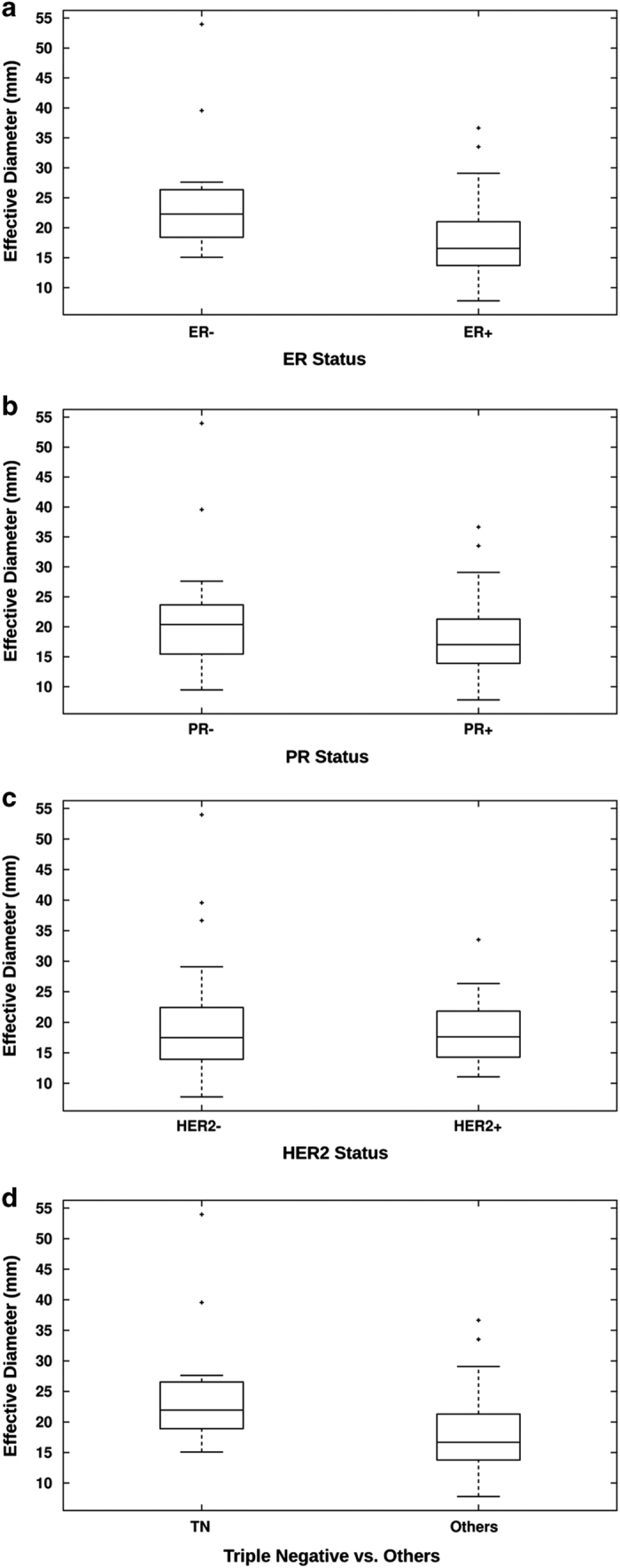
Relationship between MRI phenotype of size (effective diameter) and receptor status (**a**: ER; **b**: PR; **c**: HER2; **d**: TN). The *P*-values from the Mann–Whitney *U*-test are 0.001, 0.14, 1.0, and 0.006 for ER, PR, HER2, and TN, respectively. ER, estrogen receptor; HER2, human epidermal growth factor receptor 2; MRI, magnetic resonance imaging; PR, progesterone receptor; TN, triple negative.

**Figure 3:**
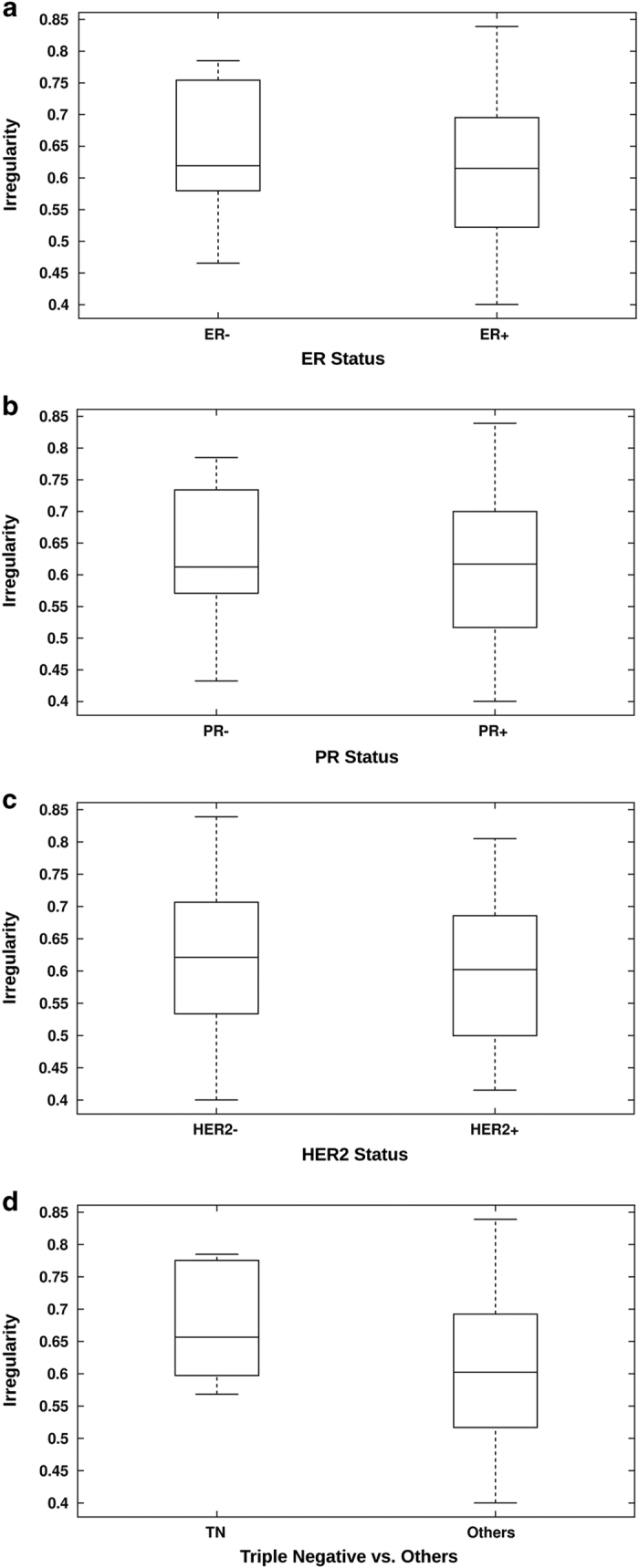
Relationship between MRI phenotype of lesion shape (irregularity) and receptor status (**a**: ER; **b**: PR; **c**: HER2; **d**: TN).The *P*-values from the Mann–Whitney *U*-test are 0.23, 0.43, 0.36, and 0.03 for ER, PR, HER2, and TN, respectively. ER, estrogen receptor; HER2, human epidermal growth factor receptor 2; MRI, magnetic resonance imaging; PR, progesterone receptor; TN, triple negative.

**Figure 4:**
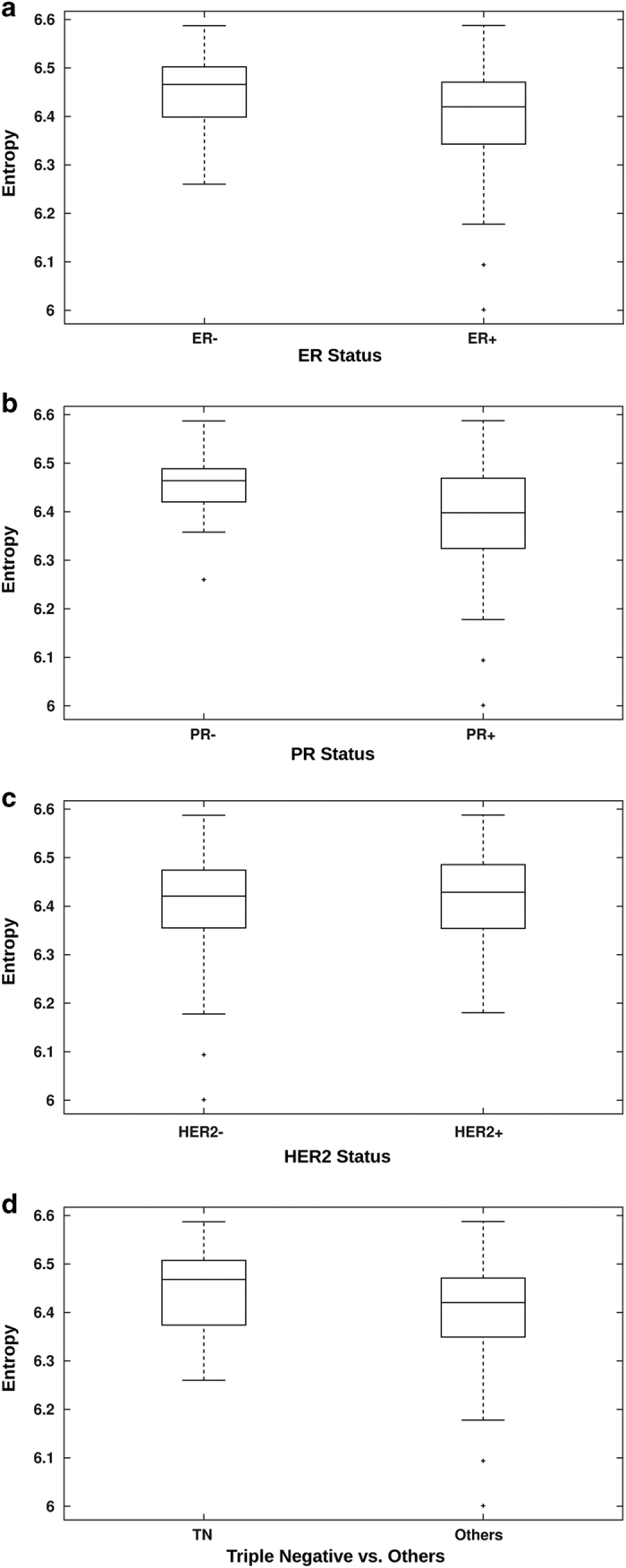
Relationship between MRI phenotype of enhancement texture (entropy) and receptor status (**a**: ER; **b**: PR; **c**: HER2; **d**: TN). The *P*-values from the Mann–Whitney *U*-test are 0.08, 0.03, 0.93, and 0.13 for ER, PR, HER2, and TN, respectively. ER, estrogen receptor; HER2, human epidermal growth factor receptor 2; MRI, magnetic resonance imaging; PR, progesterone receptor; TN, triple negative.

The box plots of MRI phenotypes of size, shape, and enhancement texture based on the receptor status (ER, PR, HER2, TN) are shown in Figures 2, 3, 4. As shown in Table 1, which gives the average values and standard deviations, the ER-negative cases tended to have larger lesion size, were more irregular in shape, and were more heterogeneous in contrast enhancement texture than those of the ER+ cases. The PR− cases tended also, on average, to be larger in size, more irregular in shape, and more heterogeneous in contrast enhancement than those of the PR+ cases. The TN cases also showed similar trends as compared with non-TN cases. Mann–Whitney test results showed the differences in the mean values of some MRI phenotypes of size and enhancement texture across the molecular classifications (Table 1; Figures 2, 3, 4).

**Figure 5:**
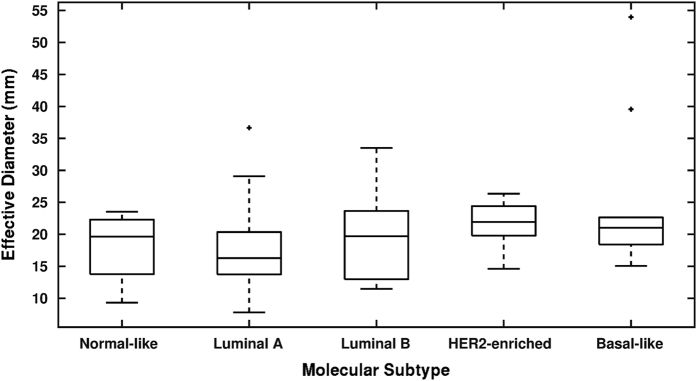
Relationship between the MRI phenotype of size (effective diameter) and the molecular subtypes. Shown is a statistically significant trend between size and molecular subtype (*P*-value of 0.01 from the Kendall test). HER2, human epidermal growth factor receptor 2; MRI, magnetic resonance imaging.

**Figure 6:**
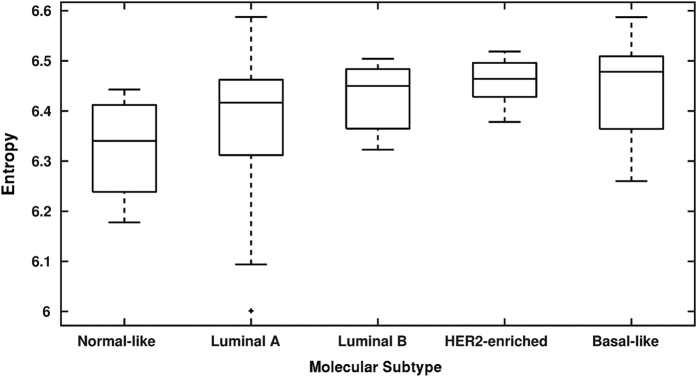
Relationship between the MRI phenotypes of enhancement texture (entropy) and the molecular subtypes. The enhancement texture is calculated at the first-post-contrast MR image thus quantitatively characterizing the heterogeneous uptake of contrast within the tumor. Shown is a statistically significant trend between entropy and molecular subtype (*P*-value of 0.006 from the Kendall test). HER2, human epidermal growth factor receptor 2; MRI, magnetic resonance imaging.

**Figure 7:**
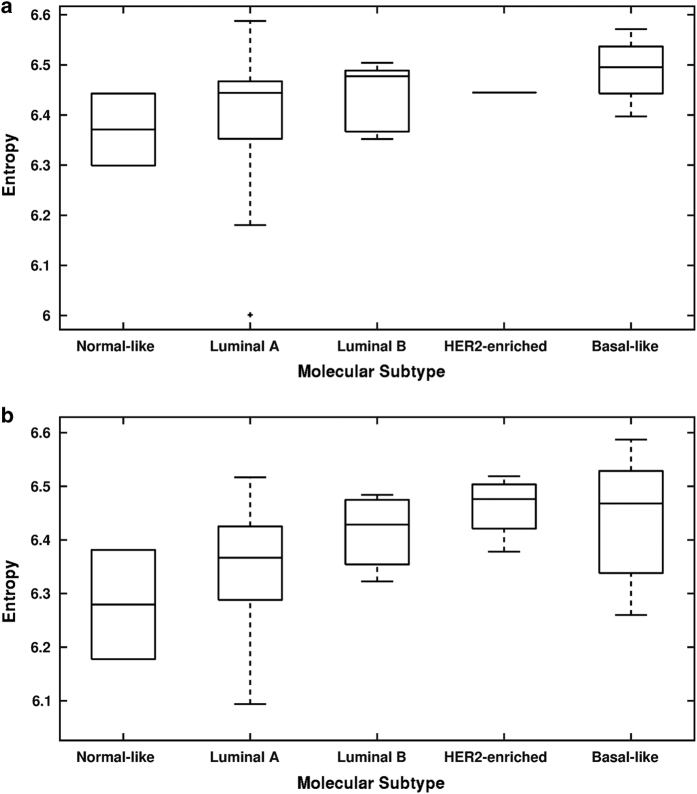
Relationship between the MRI phenotypes of enhancement texture (entropy) and the molecular subtypes based on size strata (**a**: lesion size⩽2 cm; **b**: lesion size >2 cm to ⩽5 cm). Shown are the statistically significant trends between entropy and molecular subtype (*P*-values of 0.04 and 0.02, respectively, from the Kendall tests). HER2, human epidermal growth factor receptor 2; MRI, magnetic resonance imaging.

From the Kendall test, examination of the relationship between the MRI phenotype of size (effective diameter) and cancer subtype demonstrated a statistically significant positive trend for size with molecular subtypes (*P*-value=0.01; Figure 5). Similarly, a positive trend was also observed between the MRI phenotype of enhancement texture (entropy) and cancer subtype, with the Kendall test indicating statistical significance (*P*-value=0.006; Figure 6). Results indicate that the more aggressive cancers are likely to be larger in size with more heterogeneity in their contrast enhancement. Even after controlling for tumor size, a similar statistically significant trend was observed within each size group (*P*=0.04 for lesions size ⩽2 cm; *P*=0.02 for lesions size >2 to ⩽5 cm) as with the entire data set for the relationship between MRI phenotype of enhancement texture (entropy) and the cancer subtype (Figure 7).

## Discussion

The results from this study indicate that quantitative MRI analysis shows promise as a means for high-throughput image-based phenotyping in the discrimination of breast cancer subtypes. Note that in this pilot study, we were interested in only the performance of the MRI phenotypes, thus we assessed the performance of the image-based phenotypes without investigating the impact on radiologist assessment, with or without the combination of genetic information derived from pathology.

Our results indicate that ER-negative cases may be larger, more irregular in shape, more heterogeneous, and have a faster contrast uptake rate than those of ER-positive cases (Table 1). Similar observations were also reported by Chen *et al.*^31^ in a correlation study between ER status and breast MRI radiomics. These phenotypes reiterate prior literature, capturing on imaging that ER− tumors have higher microvessel density,^32^ higher levels of vascular endothelial growth factor,^33^ and higher proliferative activity.^33^ PR-negative cases also tended to be larger, more irregular in shape, more heterogeneous, and have a faster contrast uptake than those of PR-positive cases—though not statistically significant (Table 1). These observations may visually convey the existing evidence that PR− cancers tend to have high growth factor signaling.^34^ In other words, PR loss (and correlative imaging phenotypes) may serve as a surrogate marker for excessive growth factor receptor activation.

TN cases, on average, tended to be larger, more irregular in shape, more heterogeneous, and have a faster contrast uptake rate as compared with all the other cases (Table 1). Similar observations were reported by Agner *et al.*^22^ who showed more lesion heterogeneity in TN breast cancers relative to non-TN cancers. Youk *et al.*^23^ reported that larger tumor size was significantly associated with TN breast cancer. Finally, basal-like cancers appeared more heterogeneous in enhancement texture patterns (Figures 6 and 7). The majority of basal-like cancer cases (9 out of 10) in this study were TN cases (Table 2). Basal-like breast cancer is defined not only by the absence of ER, PR and HER2 receptors, but also by the over expression of oncogenes that favor cell proliferation,^35^ suggesting why basal-like tumors tend to be larger in size. We posit that imaging phenotypes like heterogeneity and contrast enhancement, evident on dynamic contrast-enhanced breast MRI, capture and convey pathophysiologic characteristics like proliferation and angiogenesis and have the further potential to provide clues to more accurate prognosis and optimal treatment.

It is interesting to note that enhancement texture (especially heterogeneity) emerged as an important discriminatory indicator. As the enhancement texture is calculated at the first post-contrast time point of the MR image, this enhancement texture phenotype quantitatively characterizes the heterogeneous nature of the contrast uptake within a breast tumor.^36^ The larger the enhancement texture entropy, the more heterogeneous the tumor. It is important to note that, based on our analysis stratified by tumor size, we believe that molecular subtype is related to the heterogeneous nature of contrast uptake within the breast tumor and not just dependent on lesion size. Waugh *et al.*^37^ also performed texture analysis in classification of primary breast cancer, and the similar observation was reported that HER2-enriched and TN cancers showed a significant increase in entropy value relative to luminal A and luminal B cancers. A study by Grimm *et al.*^25^ showed that imaging features can predict luminal A and luminal B molecular subtypes, but not HER2-enriched and basal-like types. The evidence from our and others’ work indicate that further study is warranted in order to better understand the relationships between enhancement texture and molecular subtypes.

There are several studies^19,20,24,29,30^ in the literature that have related breast MRI kinetics to cancer subtype. Bhooshan *et al.*^19,20^ studied 353 MRI cases and correlated breast MRI radiomics features to non-invasive and invasive cancer types. Mazurowski *et al.*^24^ performed radiogenomic analysis on a subset of 48 cases from TCIA to determine whether enhancement dynamics could predict the luminal B subtype. A study by Yamaguchi *et al.*^29^ investigated the connection between kinetic curve pattern and molecular subtypes. Blaschke *et al.*^30^ reported that HER2-enriched tumors have a larger volume of rapid early uptake compared with other molecular types. In our study, the faster contrast uptake was observed in ER−, PR−, and TN cancers relative to ER+, PR+ and non-TN cancers. All these studies indicate that enhancement kinetics may reflect underlying tumor biological characteristics (*in vivo*) and warrant further research to understand these phenomena more fully.

These are several limitations in our study. The MR images used in this study were acquired more than 10 years ago by four different institutions, which may have had different acquisition protocols, different weight-based dosing regimen for contrast agents, and different time resolution of post-contrast sequences, and thus, these images may not reflect current MRI technology, which has advanced substantially during the past decade. Given higher signal-to-noise, improved spatial resolution, and more standardized imaging acquisition protocols, we would expect to see even more associations with molecular subtype. The large majority of cases came from only two sites. This has potential to introduce site bias. Thus, we performed leave-one-case-out cross validation to minimize the bias, and not to overestimate the performance on this limited data set. In addition, our patient population was predetermined by TCGA inclusion criteria. Cases included in the TCGA data set, in general, required surgical resection of at least 200–300 mg of tissue for deep genomic and proteomic analyses and all were acquired prior to any treatment. Breast MRIs were included only if they were performed prior to surgical resection. Thus, our results can only be generalized to this population. Another limitation of our study was that the patient sample was relatively small since currently breast MR images are not available for most of the TCGA breast cancer cases. Database bias was limited, however, by performing a cross validation for each molecular classification assessment (such as distinguishing between ER+ and ER− cases). Also, for some of the classification tasks the subtype prevalence was rather skewed (e.g., 11 TN versus 80 non-TN cancers) and hence results of this pilot study are somewhat preliminary. Despite all these limitations, the TCGA data set is still the largest publicly available data set for radiogenomic breast cancer research.

Future work will include studies on a larger clinical data set to verify the results from this preliminary study and further assess the role of the MRI phenotypes in combination with human visual clinical assessment and/or genomic information. It is our hope that, ultimately, merging imaging phenotypes with genomic data may lead to improved predictors.

We have demonstrated that there are statistically significant associations between image-based tumor phenotypes and receptor status. In addition, a statistically significant trend was observed between enhancement texture and intrinsic molecular subtypes of normal-like, luminal A, luminal B, HER2-enriched, and basal-like. The results from this study have the potential to provide insight into the underlying tumor biology including tumor heterogeneity. Also, using imaging phenotypes to identify molecular subtypes may aid in clinical diagnosis and treatment planning. Such imaging data and associated radiomics may serve as a “virtual biopsy”, which is non-invasive, includes the entire tumor, and is repeatable. In summary, computer-extracted MRI phenotypes show promise as a means for high-throughput discrimination of breast cancer subtypes and may yield a quantitative predictive signature for advancing precision medicine.

## Materials and methods

### Study population

Data analyzed in this study had been collected by the NCI under Institutional Review Board-approved HIPAA compliant protocols. We had access to de-identified data only, including the MR image data and the molecular classifications from the TCGA breast invasive carcinoma initiative for which breast cancer cases were solicited from cancer centers across the United States. The clinical, pathology, and molecular classification data were downloaded from the TCGA data portal using the software ‘TCGA-Assembler’.^38^ In addition, the classification of cancer subtypes was obtained from RNA sequencing gene expression data determined in the Perou lab at University of North Carolina, yielding normal-like, luminal A, luminal B, HER2-enriched, and basal-like subtype calls.^39,40^

In the TCIA, a MRI data set of 108 examinations was available at the time of this study (http://www.cancerimagingarchive.net). To reduce potential image acquisition variation, we analyzed only breast MRI studies that were similar in acquisition and technique, namely, MRIs that were acquired on a 1.5 Tesla magnet strength using GE (GE Medical Systems, Milwaukee, WI, USA) scanners and protocols; a total of 93 cases. One case with missing images in the dynamic sequence and one case without genotyping data were excluded from the study. Thus, a study database of 91 breast cancer patients resulted (Table 2) with images contributed by four institutions: Memorial Sloan Kettering Cancer Center, Mayo Clinic, University of Pittsburgh Medical Center, and Roswell Park Cancer Institute. The cases contributed by each institution were 9 (date range 1999–2002), 5 (1999–2003), 46 (1999–2004), and 31 (1999–2002), respectively. The average age of the 91 patients was 53.6 years with a standard deviation of 11.5 years and a range from 29 to 82 years with a median of 53 years. All patients had confirmed breast cancer. Out of the 91 invasive breast carcinoma cases, 79 were ductal carcinoma, 10 were lobular carcinoma, and 2 were mixed. The MRI examinations had been performed for cancer staging purpose prior to any treatment. In this paper, we will refer to breast cancer cells with or without the investigated hormone receptors as ER+, ER−, PR+, PR−, HER2+, and HER2−, respectively. For 84 of the 91 cases in the study, gene expression classifications of ‘intrinsic’ cancer molecular subtypes normal-like, luminal A, luminal B, HER2-enriched, and basal-like were also available. Note that this TCGA/TCIA data set of breast cancer cases is significant (although limited in size) since it represented the largest publicly available set of breast MRIs that had corresponding clinical data, pathologic data, and genomic data.

### Image data

MRIs had been acquired with a standard double breast coil on a 1.5T GE whole-body MRI system (GE Medical Systems). Only T1-weighted dynamic contrast-enhanced MR images were used in this study. The imaging protocols included one pre- and three to five post-contrast images obtained using a T1-weighted three-dimensional spoiled gradient echo sequence with a gadolinium-based contrast agent. In-plane resolution ranged from 0.53 to 0.86 mm, and spacing between slices ranged from 2 to 3 mm.

### Radiologist review of MR images

Each breast MRI examination was reviewed, using ClearCanvas^41,42^ (http://www.radiology.northwestern.edu/research/areas-of-research/Imaging-Informatics-home/Software%20Tools.html) (ClearCanvas, Toronto, ON, Canada), by 3 of 11 expert board-certified breast radiologists who were blinded to outcomes and who each annotated the cases independently to yield information on the approximate center location of each breast cancer. The breast imaging experience of the 11 readers was 17 years on average with a standard deviation of 6 years and a range from 4 to 25 years with a median of 15 years. For the reported study, the only input to the computer was the radiologist-determined tumor center location (by consensus) on MRI.

### Extraction of MRI-based tumor phenotypes

Given the approximate tumor center location on the MRI, each primary breast tumor was automatically segmented in three-dimensional from the surrounding parenchyma using a fuzzy c-means clustering-based method, described in detail elsewhere.^43^

A total of 38 mathematical descriptors of the breast tumors were extracted from the computer-derived tumor segmentations (Supplementary Appendix Table A1). These CEIPs can be divided into six MRI phenotypic categories describing (a) size, (b) shape, (c) morphology, (d) enhancement texture, (e) kinetic curve assessment, and (f) enhancement-variance kinetic features.^36,44–49^

The tumor size CEIPs yield information on the tumor effective diameter, volume, maximum linear size, and surface area. Effective diameter is the diameter of a sphere with the same volume as the tumor. Maximum linear size is the maximum distance between any two voxels in the three-dimensional tumor. Both effective diameter and maximum linear size were calculated from computer-segmented three-dimensional breast tumors. Note that the standard clinically-measured tumor size, as measured by the radiologists, is the largest length on MRI, and it was determined by radiologists using ClearCanvas software on a two-dimensional MR slice. For multi-focal lesions, only the primary/largest lesion was measured for tumor size. The correlation coefficients between clinical-measured tumor size and four computer size phenotypes ranged from 0.63 (surface area) to 0.79 (effective diameter). The computer-extracted size phenotype, measuring effective diameter, correlated the most with the radiologist’s size measure, and thus, was used to group the tumors into different size groups based on TNM staging criteria.^50^ Tumor CEIPs of shape include sphericity and irregularity.

Morphological CEIPs combined tumor shape and margin characteristics, and include CEIPs of margin sharpness, variance of margin sharpness, and variance of radial gradient histogram, which are used to assess tumor spiculation.^44^

Enhancement texture tumor CEIPs characterize the tumor textural properties of the contrast-enhanced tumors on the first post-contrast images, i.e., the heterogeneity of the uptake, and thus potentially the heterogeneity of angiogenesis within the tumor. These texture CEIPs were calculated from the gray-level co-occurrence matrix, with extracted features including image homogeneity, image linearity, gray-level dependence, local image variation, and randomness.^36,45^

Kinetic curve assessment tumor CEIPs characterize the physiological process of the uptake and washout nature of the contrast agent in a breast tumor during the dynamic imaging series and were calculated from the kinetic curve obtained from the most enhancing voxels within the tumor. The kinetic features include maximum contrast enhancement, time to peak, uptake rate, washout rate, curve shape index, and signal enhancement ratio.^19,46,48,49^ Enhancement–variance kinetics tumor CEIPs characterize the time course of the spatial variance of the enhancement within a breast tumor, and those extracted features include maximum variance of enhancement, time to peak, variance increase rate, and variance decrease rate.^47^

### Determination of the clinical receptor status

ER status and PR status of our data set samples were obtained from the TCGA data portal using the open-source software ‘TCGA-Assembler’.^38^ HER2 status of the samples was obtained from TCGA benchmark paper for BRCA published in Nature,^13^ which generated a HER2 call for a sample based on its molecular data if its clinical HER2 call was missing. The summary regarding the receptor statuses for the patients used in this study is listed in Table 2.

### Determination of the breast cancer molecular subtypes

The determination of breast cancer intrinsic subtypes was obtained from the Perou lab at the University of North Carolina, yielding normal-like, luminal A, luminal B, HER2-enriched, and basal-like cells.^39,40^ Cancer subtypes were determined using the PAM50 classifier.^40^ The training set used in the PAM50 algorithm is comprised of 50% ER+ samples. However, in the TCGA data set, there were ~80% ER+ samples. In order to normalize TCGA data similarly to the prevalence of ER in the PAM50 training set, TCGA RNA sequencing data was sub-sampled for a group of cases with 50% ER+ that included 157 ER− and 157 randomly selected ER+ cases with a freeze date of 7 September 2012. The median gene expression value for the subset was determined and applied to the full TCGA data set prior to running the PAM50 algorithm to determine the cancer subtypes.^40^

### Statistical analysis of the prediction of molecular classifications and cancer subtypes

We assessed the performances of the MRI CEIPs in four classification tasks based on the immunohistochemistry molecular classifications: (i) estrogen (ER+ versus ER−), (ii) progesterone (PR+ versus PR−), (iii) HER2 (HER2+ versus HER2−), and (iv) TN (TN versus others) receptor status. Receiver operating characteristic (ROC) analysis was used to assess the performance of each of the four classification tasks. The ROC analysis employed the semi-parametric ‘proper’ binormal ROC model^51–53^ using either the CEIPS or the linear classifier output scores as the decision variable, with the AUC as the figure of merit. Statistical significance was assessed with respect to the baseline of random guessing (AUC=0.5). In addition, Mann–Whitney *U-*tests were conducted to further investigate the differences in the CEIPs between the molecular classifications.^54^

To obtain an approximately unbiased estimate of the classification accuracy of the predictive models, we conducted a single leave-one-case-out cross validation including both forward and backward feature selection and tumor classification. That is, within each iteration of the leave-one-case-out cross validation, tumor CEIPs were selected and subsequently used as input to a linear discriminant analysis classifier to yield a classifier output score for the test case (tumor). The phenotypic combinations selected in each iteration of the cross validation were tabulated, and after all iterations, the most frequently selected combination (i.e., phenotypic signature) was reported for each molecular classification task.

A non-parametric Kendall test^55^ was conducted between the CEIPs and the molecular (gene expression) cancer subtypes: normal-like, luminal A, luminal B, HER2-enriched, and basal-like. The molecular subtypes were ordered as indicated based on the survival data reported by other studies^56–58^ and thus were treated as ordinal variables in the association study. Normal-like breast tumors are usually small and tend to have very favorable prognosis.^57^ Luminal A breast cancers are associated with favorable prognosis, with fairly high survival rates and low recurrence rates; the 5-year survival rate is more than 80%.^56,58^ Women with luminal B tumors are often diagnosed at a younger age than those with luminal A cancers and have poorer prognosis than luminal A tumors with five-year survival rates of approximately 40%.^56,58^ HER2-enriched breast tumors are generally intermediate- to high-grade tumors with a 5-year survival rate of 31%.^56^ Basal-like tumors tend to occur more often in younger women and African-American women, and are often aggressive and have the worst prognosis among the breast cancer subtypes.^56,58^ The Kendall coefficient tau-b was computed to indicate the association between CEIPs and molecular subtypes. Moreover, since size is an important prognostic factor, we also investigated the relationship between image-based phenotypes and molecular subtypes within each lesion size strata based on TNM staging criteria, in which the data set was divided into three size groups based on effective diameters: ⩽2 cm (T1); >2 to ⩽5 cm (T2); or >5 cm (T3). There were 51 cases with an effective diameter less or equal to 2 cm, 32 with tumor size between 2 and 5 cm, and only 1 case with a tumor larger than 5 cm.

The statistical significance threshold was set at a *P*-value of 0.05 to indicate statistical significance for a single comparison. However, for multiple comparisons, the Holm’s *t*-test^59^ for multiple comparisons of significance was employed with evaluate the statistical significance using an overall significant level *α*^T^=0.05. All in-house analysis routines were written in MatLab (v.8.0, MathWorks, Natick, MA, USA).

## Funding

This research was funded in part by the University of Chicago Dean Bridge Fund, and by NCI U01-CA195564, U24-CA143848-05, P50-CA58223 Breast SPORE program, and the Breast Cancer Research Foundation.

## Figures and Tables

**Table 1:** Results from the Mann–Whitney *U*-test indicating association between MRI phenotypes and molecular classifications for phenotypes shown in Figures 2, 3, 4

*Classification task*	*Number of tumors*	*MRI phenotype*	*Mean value (s.d.) positive versus negative*	P*-value*	*Significance level (*α^*T*^*=0.05)*
ER+ versus ER−	91 (77 vs. 14)	Effective diameter	17.6 mm (5.6) vs. 24.8 mm (10.4)	0.001^a^	0.0167
		Irregularity	0.61 (0.11) vs. 0.65 (0.11)	0.23	0.05
		Entropy	6.40 (0.11) vs. 6.45 (0.09)	0.08	0.025
PR+ versus PR−	91 (72 vs.19)	Effective diameter	18.0 mm (5.6) vs. 21.6 mm (10.5)	0.14	0.025
		Irregularity	0.61 (0.11) vs. 0.63 (0.10)	0.43	0.05
		Entropy	6.39 (0.11) vs. 6.45 (0.07)	0.03	0.0167
HER2+ versus HER2−	91 (19 vs. 72)	Effective diameter	18.4 mm (5.7) vs. 18.8 mm (7.3)	1.0	0.05
		Irregularity	0.59 (0.12) vs. 0.62 (0.11)	0.36	0.0167
		Entropy	6.41 (0.10) vs. 6.40 (0.11)	0.93	0.025
TN versus others	91 (11 vs. 80)	Effective diameter	17.8 mm (5.6) vs. 25.6 (11.5)	0.006^a^	0.0167
		Irregularity	0.60 (0.11) vs. 0.68 (0.09)	0.03	0.025
		Entropy	6.40 (0.10) vs. 6.45 (0.10)	0.13	0.05

Abbreviations: ER, estrogen receptor; HER2, human epidermal growth factor receptor 2; MRI, magnetic resonance imaging; PR, progesterone receptor; TN, triple negative.

a Indicates statistical significance was achieved after correction for multiple comparisons.

**Table 2:** A cross tabulation of receptor status and intrinsic molecular subtype

*Molecular subtype (research values)*	*Total*	*Receptor status*							
		*ER+*	*ER−*	*PR+*	*PR−*	*HER2+*	*HER2−*	*Triple negative*	*Others*
All cases	91	85% (77/91)	15% (14/91)	79% (72/91)	21% (19/91)	21% (19/91)	79% (72/91)	12% (11/91)	88% (80/91)
Normal-like	5% (4/84)	100% (4/4)	0% (0/4)	100% (4/4)	0% (0/4)	25% (1/4)	75% (3/4)	0% (0/4)	100% (4/4)
Luminal A	65% (55/84)	100% (55/55)	0% (0/55)	95% (52/55)	5% (3/55)	15% (8/55)	85% (47/55)	0% (0/55)	100% (55/55)
Luminal B	12% (10/84)	100% (10/10)	0% (0/10)	80% (8/10)	20% (2/10)	40% (4/10)	60% (6/10)	0% (0/10)	100% (10/10)
HER2-enriched	6% (5/84)	60% (3/5)	40% (2/5)	40% (2/5)	60% (3/5)	100% (5/5)	0% (0/5)	0% (0/5)	100% (5/5)
Basal-like	12% (10/84)	10% (1/10)	90% (9/10)	10% (1/10)	90% (9/10)	10% (1/10)	90% (9/10)	90% (9/10)	10% (1/10)
Not determined	7	57% (4/7)	43% (3/7)	71% (5/7)	29% (2/7)	0% (0/7)	100% (7/7)	29% (2/7)	71% (5/7)

Abbreviations: ER, estrogen receptor; HER2, human epidermal growth factor receptor 2; PR, progesterone receptor.

## Notes

Supplementary Appendix Table A1
