# Supplementary material for: Quantitative MRI radiomics in the prediction of molecular classifications of breast cancer subtypes in the TCGA/TCIA data set
Source: NPJ Breast Cancer. 2016 May 11;2:16012–. doi: 10.1038/npjbcancer.2016.12 (PMC5108580; doi:10.1038/npjbcancer.2016.12)
Supplement: Supplementary Appendix Table A1 [file npjbcancer201612-s1.doc]

**APPENDIX**

Table A1. Definition of computer-extracted image radiomics phenotypes

| **Radiomic category** | **Image phenotype** | **Description** |
| --- | --- | --- |
| **Size** | Volume (mm^3^) | Volume of lesion |
|  | Effective diameter (mm) | Diameter of a sphere with the same volume as the lesion |
|  | Surface area (mm^2^) | Lesion surface area |
|  | Maximum linear size (mm) | Maximum distance between any two voxels in the lesion |
| **Shape** | Sphericity | Similarity of the lesion shape to a sphere |
|  | Irregularity | Deviation of the lesion surface from the surface of a sphere |
|  | Surface area/volume (1/mm) | Ratio of surface area to volume |
| **Morphology** | Margin sharpness | Mean of the image gradient at the lesion margin |
|  | Variance of margin sharpness | Variance of the image gradient at the lesion margin |
|  | Variance of radial gradient histogram | Degree to which the enhancement structure extends in a radial pattern originating from the center of the lesion |
| **Enhancement Texture** | Contrast | Local image variations |
|  | Correlation | Image linearity |
|  | Difference entropy | Randomness of the difference of neighboring voxels’ gray-levels |
|  | Difference variance | Variations of difference of gray-levels between voxel-pairs |
|  | Angular second moment (Energy) | Image homogeneity |
|  | Entropy | Randomness of the gray-levels |
|  | Inverse difference moment | Image homogeneity |
|  | Information measure of correlation (IMC) 1 | Nonlinear gray-level dependence |
|  | Information measure of correlation (IMC) 2 | Nonlinear gray-level dependence |
|  | Maximum correlation coefficient | Nonlinear gray-level dependence |
|  | Sum average | Overall brightness |
|  | Sum entropy | Randomness of the sum of gray-levels of neighboring voxels |
|  | Sum variance | Spread in the sum of the gray-levels of voxel-pairs distribution |
|  | Sum of squares (Variance) | Spread in the gray-level distribution |
| **Kinetic Curve Assessment** | Maximum enhancement | Maximum contrast enhancement |
|  | Time to peak (s) | Time at which the maximum enhancement occurs |
|  | Uptake rate (1/s) | Uptake speed of the contrast enhancement |
|  | Washout rate (1/s) | Washout speed of the contrast enhancement |
|  | Curve shape index | Difference between late and early enhancement |
|  | Enhancement at first post-contrast time point | Enhancement at first post-contrast time point |
|  | Signal enhancement ratio | Ratio of initial enhancement to overall enhancement |
|  | Volume of most enhancing voxels (mm^3^) | Volume of the most enhancing voxels |
|  | Total rate variation (1/s^2^) | How rapidly the contrast will enter and exit from the lesion |
|  | Normalized total rate variation (1/s^2^) | How rapidly the contrast will enter and exit from the lesion |
| **Enhancement-Variance Kinetics** | Maximum variance of enhancement | Maximum spatial variance of contrast enhancement over time |
|  | Time to peak at maximum variance (s) | Time at which the maximum variance occurs |
|  | Enhancement variance increasing rate (1/s) | Rate of increase of the enhancement-variance during uptake |
|  | Enhancement variance decreasing rate (1/s) | Rate of decrease of the enhancement-variance during washout |
